# Phototherapy Alone or Combined with Adjuvant Drugs for Neonatal Hyperbilirubinemia: A Systematic Review and Network Meta-Analysis

**DOI:** 10.3390/children13040573

**Published:** 2026-04-20

**Authors:** Qiang Fei, Huazi Liu, Xinning Wang, Tianming Yuan

**Affiliations:** Department of Neonatology, Children’s Hospital, School of Medicine, Zhejiang University, National Clinical Research Center for Child and Adolescents’ Health and Disease, Hangzhou 310000, China; feiqiang@zju.edu.cn (Q.F.); liuhuazi@zju.edu.cn (H.L.); xinningwang@zju.edu.cn (X.W.)

**Keywords:** neonate, jaundice, treatment, randomized controlled trials

## Abstract

**Highlights:**

**What are the main findings?**

- Adjunctive therapies combined with phototherapy were associated with greater bilirubin reduction than phototherapy alone.
- Fibrates and UDCA ranked higher in the analysis for improving bilirubin reduction and facilitating the resolution of jaundice, while probiotics, zinc, and agar showed modest effects, and phenobarbital showed no clear benefit.

**What are the implications of the main findings?**

- This analysis provides a comparative overview of adjunctive therapies in resolving jaundice. Further well-designed RCTs are required to establish efficacy and safety before clinical use.

**Abstract:**

**Objectives**: Neonatal hyperbilirubinemia is a common disease in the neonatal period. In this meta-analysis, we aim to evaluate the efficacy of adjuvant drugs combined with phototherapy in the treatment of neonatal hyperbilirubinemia. **Methods**: Randomized controlled trials (RCTs) published before September 2025 were searched from PubMed, Embase, Web of Science, and the Cochrane Library. A Bayesian random-effects network meta-analysis was performed to calculate mean differences and 95% confidence intervals. Interventions were ranked using the surface under the cumulative ranking curve (SUCRA) and probability of being the best treatment (PbBT). **Results**: Thirty-five RCTs involving 4060 neonates were included. Compared with phototherapy alone, clofibrate, ursodeoxycholic acid, fenofibrate, and calcium phosphate significantly reduced bilirubin levels and shortened admission duration. Clofibrate showed the greatest efficacy in bilirubin reduction within 48 h (SUCRA = 0.91, PbBT = 60.9%) and in shortening hospitalization (SUCRA = 0.84, PbBT = 40.83%). Probiotics, zinc, and agar exhibited relatively modest effects, while phenobarbital showed no significant benefit. **Conclusions**: Adjunctive therapies were associated with greater reductions in bilirubin levels compared with phototherapy alone. Future high-quality RCTs are needed to confirm the long-term efficacy and safety of these adjuvant therapies.

## 1. Introduction

Neonatal hyperbilirubinemia is one of the most common clinical conditions in the neonatal period, affecting approximately 50–90% of newborns within the first week of life [1,2,3]. Although jaundice is physiological in most cases, excessively elevated serum bilirubin levels can lead to severe neurological complications, including acute bilirubin encephalopathy and kernicterus [4]. Phototherapy remains the first-line treatment for neonatal hyperbilirubinemia, as it converts unconjugated bilirubin into water-soluble isomers that can be easily excreted [1,2,3]. However, phototherapy has several disadvantages, such as prolonged treatment duration, mother–infant separation, diarrhea, dehydration, temperature instability, and increased healthcare costs [5,6].

In recent years, a variety of adjuvant drugs have been studied for enhancing the effect of phototherapy, including fibric acid derivatives (such as clofibrate, fenofibrate), ursodeoxycholic acid (UDCA), probiotics (such as bifidobacterium, lactic acid bacteria), phenobarbital, agar, zinc, etc. [7,8,9,10,11,12,13,14,15,16,17,18,19,20,21,22,23,24,25,26,27,28,29,30,31,32,33,34,35,36,37,38,39,40,41]. Although the efficacy of these adjuvant drugs has been evaluated by several randomized controlled trials, the research results show significant heterogeneity. Studies have found that probiotics can significantly reduce bilirubin levels in newborns and shorten admission duration [42], but some studies have not observed significant benefits [14]. Also in phenobarbital, Shah Farhat et al. [12] observed that there was no significant difference between the drug combined with phototherapy and phototherapy alone.

Although various adjuvant drugs can enhance phototherapy efficacy, medication use in newborns remains challenging, especially with multiple agents. The optimal adjuvant drug for use with phototherapy remains controversial, and no comprehensive systematic review has yet compared the relative efficacy of different adjuvant therapies. Therefore, this meta-analysis aims to evaluate the efficacy of adjuvant drugs combined with phototherapy in the treatment of neonatal hyperbilirubinemia. Primary outcomes include the levels of bilirubin reduction, phototherapy duration, and admission duration. This study quantitatively compares drugs to guide clinical decisions and improve the management of neonatal hyperbilirubinemia.

## 2. Materials and Methods

### 2.1. Search Strategy

The study was conducted in accordance with the Preferred Reporting Items for Systematic Reviews and meta-analyses (PRISMA) 2020 standards [43], and it has been registered in advance on PROSPERO (CRD420251174122). We conducted a systematic search in Pubmed, Embase, Web of science and Cochrane library. The last search was conducted on 25 September 2025. The retrieval strategy is as follows: (“Infant OR Newborn”) AND (“Hyperbilirubinemia” OR”Bilirubinemia”) AND (“Phototherapy” OR “Blue Light Therapy”) AND (“randomized controlled trial” OR “randomized”) AND (“probiotics” OR “clofibrate” OR “zinc sulfate” OR “ursodeoxycholic acid” OR “calcium phosphate” OR “agar” OR “phenobarbital” OR “yinzhihuang” OR “fenofibrate”).

### 2.2. Inclusion and Exclusion Criteria

Studies were included if they met the following criteria: (1) randomized controlled trials (RCTs); (2) participants were newborns diagnosed with hyperbilirubinemia; (3) received phototherapy; (4) reported at least one of the following outcomes: phototherapy duration, admission duration, bilirubin levels at 24 or 48 h; (5) used one of the following adjuvant drugs: zinc sulfate, calcium phosphate, probiotics, agar, clofibrate, ursodeoxycholic acid, phenobarbital, Yingzhihuang oral liquid, or fenofibrate; and (6) published in English. For studies involving the same cohort, the one with the larger sample size or more comprehensive data was included. This analysis focused on drug types, not on dosage differences. Data from different dose groups of the same drug in one study were combined. The combined data were used to assess the overall efficacy.

Exclusion criteria were as follows: (1) meta-analyses, reviews, abstracts, letters, or case reports; (2) animal or basic research; (3) studies not reporting bilirubin levels, phototherapy duration, or admission duration; (4) non-randomized trials; and (5) newborns with pathological hemolysis (e.g., thalassemia, autoimmune hemolytic anemia, and hereditary spherocytosis et al.), infection, metabolic disorders, congenital anomalies, or other comorbid conditions.

### 2.3. Data Extraction

Extract the following information from all included studies: title, author, year of publication, country, gestational age, gender, initial bilirubin level, treatment, number of patients, 24 h and 48 h bilirubin levels (mean ± SD), duration of phototherapy and length of hospitalization (mean ± SD). After eliminating duplicate studies, the two researchers independently selected articles and extracted the data of the articles by reading the titles, abstracts and full texts. If the two parties have different opinions, the third evaluator shall discuss with them and make a decision.

### 2.4. Quality Evaluation

The methodological quality of the included randomized controlled trials was assessed using the Cochrane Risk of Bias tool (RoB 2.0). This tool evaluates five domains: (1) bias arising from the randomization process; (2) bias due to deviations from intended interventions; (3) bias due to missing outcome data; (4) bias in outcome measurement; and (5) bias in selection of the reported results. Each domain is rated as “low risk,” “some concerns,” or “high risk” based on the available information. The assessment was independently conducted by two reviewers, and any discrepancies were resolved through discussion with a third reviewer.

### 2.5. Statistical Methods

The primary outcomes were serum bilirubin levels at 24 and 48 h after treatment, and the secondary outcomes included the phototherapy duration and length of hospitalization. All outcomes in this study were continuous variables. The mean difference (MD) and standard deviation (SD) were used to assess the effect size between the intervention and phototherapy-only groups. A Bayesian random-effects network meta-analysis was performed using the Markov Chain Monte Carlo method to generate posterior distributions. Bayesian random-effects network meta-analysis was performed to calculate mean differences and 95% confidence intervals. The surface under the cumulative ranking curve (SUCRA) was calculated to rank the efficacy of interventions, with values ranging from 0 to 1; higher values indicate greater efficacy. The probability of being the best treatment (PbBT) was also estimated for each intervention. For treatment networks forming closed loops, consistency was assessed using node-splitting analysis and the Z-statistic (*p* < 0.05) to compare direct and indirect evidence. All analyses and visualizations were performed using R software (4.5.0) and JAGS (4.3.1)

## 3. Results

### 3.1. Selection Process

**Figure 1:**
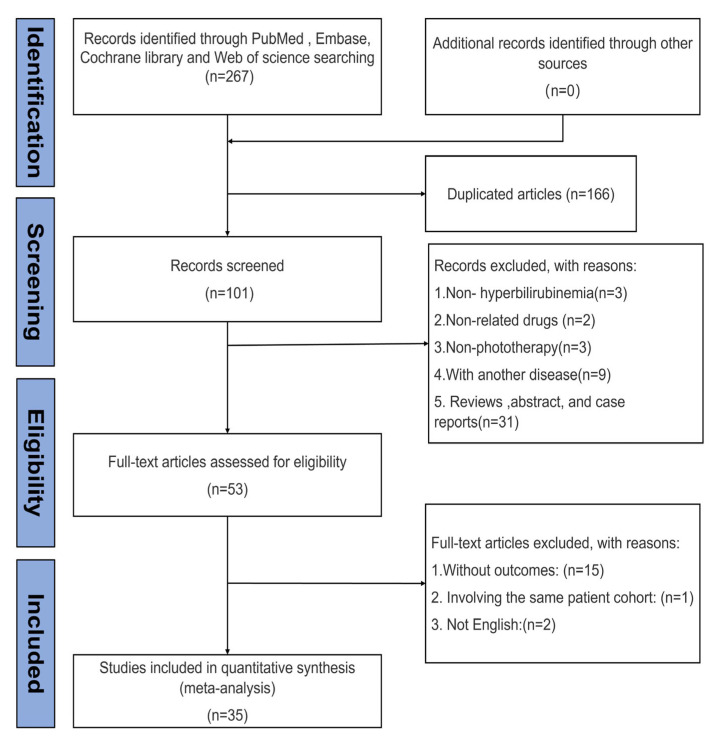
Flow diagram of selection process.

A systematic search was conducted in PubMed, Embase, Web of Science, and the Cochrane Library. A total of 267 records were identified, and 166 duplicates were removed. Based on the inclusion and exclusion criteria, 101 studies were screened, and the full texts of 53 articles were reviewed. After detailed evaluation, 18 studies were excluded—15 for not reporting relevant outcomes, one for duplicate patient data, and two for being non-English publications. Finally, 35 randomized controlled trials meeting all criteria were included in the analysis (Figure 1).

### 3.2. Data Characteristics

A total of 35 randomized controlled trials involving 4060 neonates with hyperbilirubinemia were included in the analysis (Table 1). All studies were published between 2007 and 2024, and the gestational age of the newborns ranged from 32.1 to 39.0 weeks. Most studies were conducted in Asian countries, with Iran contributing the largest number (*n* = 19), followed by India (*n* = 5), China (*n* = 3), Egypt (*n* = 3), and one study each from Bangladesh, Iraq, Turkey, Pakistan, and Jordan. Except for one trial comparing phototherapy combined with phenobarbital and probiotics versus phototherapy combined phenobarbital, all other studies used phototherapy alone or with placebo as the control group. The intervention groups included probiotics (*n* = 9), clofibrate (*n* = 7), ursodeoxycholic acid (*n* = 7), zinc (*n* = 6), fenofibrate (*n* = 5), phenobarbital (*n* = 2), agar (*n* = 1), and calcium phosphate (*n* = 1). Since this analysis focused on drug types rather than dosage differences, data from multiple dose groups of the same drug were combined and analyzed as the overall efficacy.

**Figure 2:**
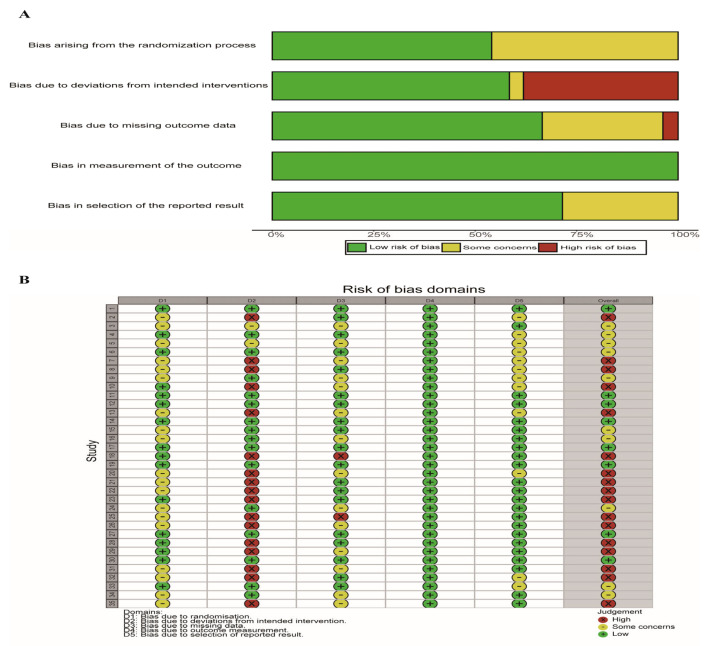
Results of risk of bias assessment. (**A**) Overall distribution of risk of bias judgments across domains. (**B**) Study-level risk of bias judgments across all domains.

The quality of the included studies was assessed using the Cochrane RoB 2.0 tool (Figure 2). The main sources of bias were deviations from intended interventions and missing outcome data. Specifically, 17 studies were rated as high risk for deviations from intended interventions, two for missing data. The remaining studies were rated as having some concerns (*n* = 10) and low risk of bias (*n* = 8).

### 3.3. Bilirubin Levels at 24 H

**Figure 3:**
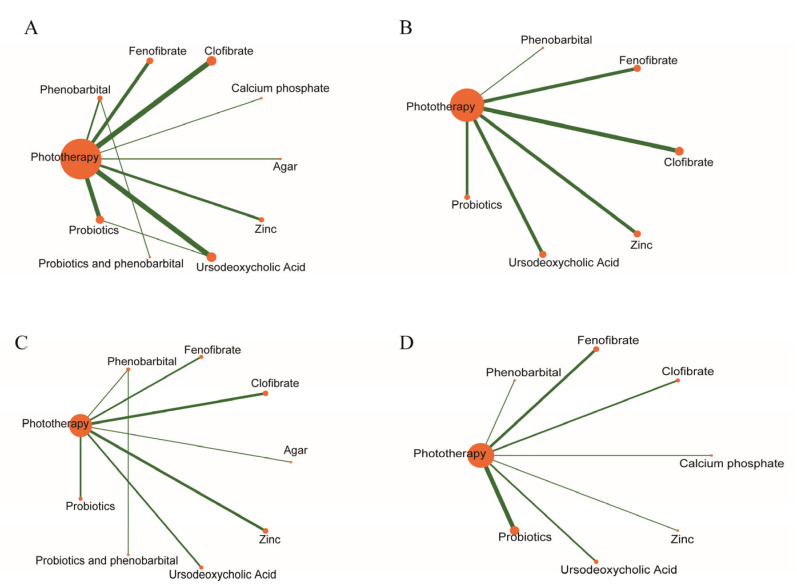
Network meta-analysis of eligible comparisons. The network interventions regarding serum bilirubin levels at 24 h (**A**). Serum bilirubin levels at 48 h (**B**), phototherapy duration (**C**), and admission duration (**D**).

**Figure 4:**
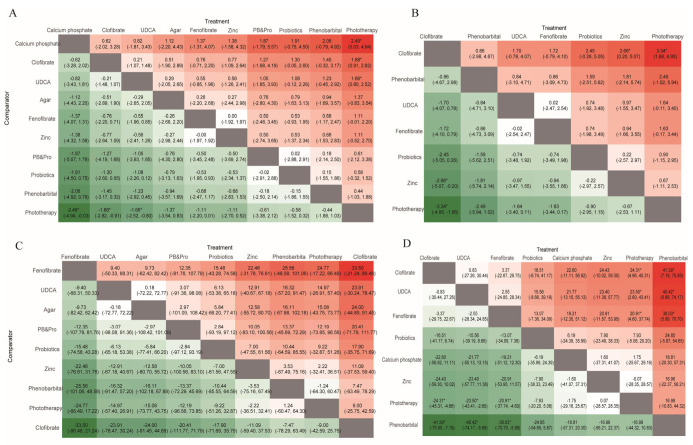
Heatmap of network meta-analysis results. The images show the pairwise comparison of different treatments based on serum bilirubin levels at 24 h (**A**), serum bilirubin levels at 48 h (**B**), phototherapy duration (**C**), and admission duration (**D**). Note: UDCA means ursodeoxycholic acid, PB&Pro means phenobarbital and probiotic. * notes *p* < 0.05.

**Figure 5:**
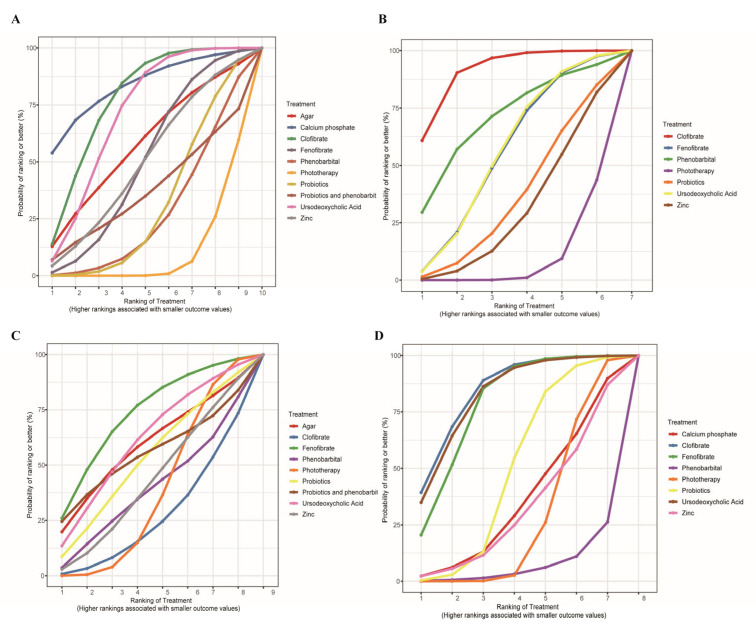
Surface under the cumulative ranking (SUCRA) curves. SUCRA curves regarding serum bilirubin levels at 24 h (**A**), serum bilirubin levels at 48 h (**B**), duration (**C**), and admission duration (**D**).

**Figure 6:**
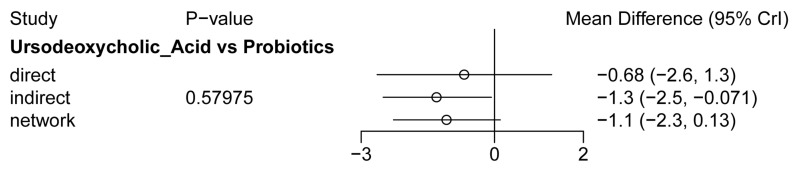
Node-splitting analysis between direct and indirect evidence.

Twenty-eight studies reported serum bilirubin levels at 24 h after treatment. The evidence network is shown in Figure 3A. The evaluated adjuvant drugs included phenobarbital, fenofibrate, clofibrate, calcium phosphate, agar, zinc, ursodeoxycholic acid, probiotics, and the combination of phenobarbital with probiotics. Compared with phototherapy alone, phototherapy combined with calcium phosphate (MD = −2.49, 95% CI: −4.94 to −0.03), clofibrate (MD = −1.88, 95% CI: −2.82 to −0.91), and UDCA (MD = −1.66, 95% CI: −2.52 to −0.80) significantly reduced bilirubin levels within 24 h (Figure 4A). The corresponding SUCRA values were 0.84, 0.78, and 0.71, respectively (Figure 5A and Table 2). Among them, calcium phosphate showed the highest probability of being the most effective treatment (PbBT = 53.89%) (Table 2). In contrast, phototherapy alone had the lowest SUCRA (0.10) and PbBT (0.00%) (Table 2), indicating the weakest effect. For the comparison between UDCA and probiotics, which formed a closed loop, node-splitting analysis showed no significant inconsistency between direct and indirect evidence (*p* = 0.58) (Figure 6).

### 3.4. Bilirubin Levels at 48 H

Twenty-one studies reported serum bilirubin levels within 48 h after treatment. The evidence network is shown in Figure 3B. The evaluated adjuvant drugs included phenobarbital, fenofibrate, clofibrate, zinc, ursodeoxycholic acid, and probiotics. Compared with phototherapy alone, phototherapy combined with clofibrate significantly reduced bilirubin levels at 48 h (MD = −3.34, 95% CI: −4.95 to −1.66) (Figure 4B), with a SUCRA value of 0.91 (Figure 5B and Table 2). Clofibrate showed the highest probability of being the most effective treatment (PbBT = 60.88%) (Table 2). Consistent with the 24 h analysis, phototherapy alone had the lowest SUCRA (0.09) and PbBT (0.00%) (Table 2), indicating the weakest efficacy in reducing bilirubin within 48 h.

### 3.5. Phototherapy Duration

Fifteen studies reported the duration of phototherapy. The evidence network is shown in Figure 3C. The evaluated adjuvant drugs included phenobarbital, fenofibrate, clofibrate, agar, zinc, ursodeoxycholic acid, probiotics, and the combination of phenobarbital with probiotics. Compared with phototherapy alone, no combination showed a statistically significant reduction in phototherapy duration (Figure 4C). However, clofibrate had a high SUCRA value (0.84) (Figure 5C and Table 2), and fenofibrate showed the highest probability of being the most effective in reducing phototherapy time (PbBT = 25.9%) (Table 2). Phototherapy alone had a SUCRA of 0.38 and the lowest likelihood of being the best option (PbBT = 0.03%) (Table 2).

### 3.6. Admission Duration

Fifteen studies reported the length of admission duration. The evidence network is shown in Figure 3D. The evaluated adjuvant drugs included phenobarbital, fenofibrate, clofibrate, calcium phosphate, zinc, ursodeoxycholic acid, and probiotics. Compared with phototherapy alone, the combination with clofibrate (MD = −24.31, 95% CI: −45.31 to −4.66), UDCA (MD = −23.50, 95% CI: −43.41 to −2.60), or fenofibrate (MD = −20.91, 95% CI: −37.74 to −4.60) significantly shortened admission duration (Figure 4D). The corresponding SUCRA values were 0.84, 0.82, and 0.79, respectively (Figure 5D and Table 2). Among them, clofibrate had the highest probability of being the most effective intervention (PbBT = 40.83%) (Table 2). In contrast, phototherapy alone had the lowest PbBT (0.00%) and was least likely to be the best option (Table 2).

### 3.7. Adverse Events

Analysis of 35 RCTs revealed that 33 reported only short-term adverse reactions (Table 3), primarily mild and transient GI symptoms, rashes, or laboratory abnormalities, with no long-term data available. Meta-analysis was precluded by low event rates and inconsistent reporting. Among the five studies reporting transfusions, rates were not lower with phototherapy alone versus combination therapy. No mortality was reported. Overall, adjuvant drugs combined with phototherapy may demonstrate good safety and tolerability in neonatal jaundice.

## 4. Discussion

To our knowledge, this is the first study to comprehensively evaluate efficacy among adjuvant drugs combined with phototherapy via network meta-analysis. This study included 35 randomized controlled trials involving 4060 neonates with hyperbilirubinemia. The results show that, compared with phototherapy alone, most adjuvant drugs can promote bilirubin reduction and shorten both phototherapy duration and admission duration. In contrast, phototherapy alone consistently showed the lowest PbBT values across outcomes, suggesting relatively lower efficacy compared with combination therapy. Clofibrate, ursodeoxycholic acid, and fenofibrate demonstrated consistent therapeutic advantages across primary outcomes, while calcium phosphate only showed potential superiority in reducing bilirubin levels at 24 h. However, given the limited long-term safety data and lack of clinically meaningful endpoints, these findings should be interpreted with caution and considered hypothesis-generating rather than practice-changing.

Calcium phosphate adsorbs unconjugated bilirubin in the intestine and inhibits enterohepatic circulation [44], resulting in a significant reduction in bilirubin levels within 24 h. However, evidence supporting this effect remains limited. Ursodeoxycholic acid (UDCA), a bile acid preparation widely used for cholestatic liver disease, promotes bile flow and alleviates hepatic bile stasis [45]. In the present study, UDCA effectively reduced bilirubin levels at 24 h and shortened hospital stay, with SUCRA values of 0.71 and 0.82, respectively, consistent with previous systematic reviews [46]. Nevertheless, UDCA is primarily indicated for conjugated hyperbilirubinemia in cholestatic conditions, whereas neonatal hyperbilirubinemia is predominantly characterized by unconjugated hyperbilirubinemia. Although the biological rationale for its application in this setting remains to be fully elucidated, mechanistic studies [47] have shown that UDCA may upregulate UDP-glucuronosyltransferase 1A1 expression in the gut of neonatal mice, thereby enhancing bilirubin metabolism and reducing plasma and brain bilirubin levels. Well-designed RCTs and further exploration of underlying mechanisms are needed before UDCA can be routinely recommended.

Clofibrate provides the greatest benefit in reducing bilirubin levels at 48 h and also ranks highly in shortening phototherapy duration and admission duration. This finding is consistent with previous clinical studies [8,22,36]. Clofibrate is a peroxisome proliferator-activated receptor α agonist [48]. It enhances hepatic glucuronosyltransferase activity, bilirubin conjugation and excretion, which helps clear unconjugated bilirubin [48], while, due to increased non-cardiovascular mortality, clofibrate has been largely withdrawn from clinical use in many countries [49]. Fenofibrate shares a similar structure and demonstrates a favorable effect in lowering bilirubin levels. Although fenofibrate’s efficacy appears slightly lower than that of clofibrate, possibly due to differences in drug dosage or the developmental maturity of hepatic enzyme systems in neonates. However, data regarding the use of fibrates in children are limited. Given the lack of robust evidence on long-term safety, their use in pediatric patients should be approached with caution [50].

Probiotics, phenobarbital, zinc, and agar have shown some efficacy in certain studies, but there is no significant difference overall. Probiotics may assist by modulating intestinal flora, inhibiting β-glucuronidase activity, and reducing enterohepatic bilirubin circulation [42]. However, their effects are highly dependent on strain, dosage, and treatment duration. Phenobarbital can induce UDP glucuronosyltransferase 1A1 expression and promote bilirubin conjugation [51]. But phenobarbital has a long half—life and potential adverse effects, such as sedation and respiratory depression, which limit its widespread use in neonates [52]. Zinc and agar may reduce intestinal bilirubin recirculation through adsorption [53,54]. However, clinical evidence is limited. Larger studies are needed to confirm their efficacy.

Previous systematic reviews have primarily focused on single- or two-drug comparisons. This study is the first to apply Bayesian network meta-analysis to integrate direct and indirect evidence regarding multiple adjuvant drugs, providing a more comprehensive ranking of efficacy. A systematic review reported that clofibrate significantly shortened phototherapy and hospital stay [55], and another study found that UDCA effectively reduced bilirubin with fewer adverse effects [46]. Notably, even in our network meta-analysis, clofibrate and UDCA consistently ranked highest. Nevertheless, safety concerns necessitate caution when selecting combination regimens, and drugs with known risks, such as clofibrate, should be avoided. This is particularly relevant in neonates, in whom physiological immaturity may alter drug metabolism and increase susceptibility to adverse effects. For UDCA, fenofibrate and calcium phosphate, further high-quality RCTs are essential to confirm their therapeutic benefits and safety in neonates.

Despite the use of strict inclusion criteria and Bayesian network meta-analysis, several limitations should be noted. First, most included studies focused on short-term biochemical outcomes, such as bilirubin reduction. Clinically meaningful endpoints, including exchange transfusion and long-term safety outcomes, were rarely reported, limiting assessment of true clinical benefit. Therefore, the findings should be considered exploratory rather than practice-changing. Second, the included studies were geographically concentrated in a limited number of countries, primarily Iran, China, Egypt, and Bangladesh. This uneven distribution may introduce regional bias and limit the generalizability of the findings to other healthcare settings. In addition, many of the evaluated adjuvant agents, such as clofibrate and certain probiotic formulations, are not widely available or routinely used in many countries, further restricting the applicability of these results in broader clinical practice. Third, although most studies reported only mild and transient adverse events, the incidence of side effects may still represent an important limiting factor for the wider clinical use of these agents. The lack of standardized reporting and absence of long-term safety data make it difficult to draw firm conclusions regarding their safety profiles. Finally, the use of pharmacological adjuvants in neonates requires particular caution due to the unique physiological characteristics of this population. Immaturity of the intestinal microbiota, hepatic metabolism, and immune system may influence both drug efficacy and safety. These factors may further limit the generalizability and clinical adoption of these therapies. Future research should confirm the efficacy and safety of these drugs through multicenter, large-sample, head-to-head randomized controlled trials.

## 5. Conclusions

In summary, this study systematically integrated evidence and performed network comparisons to clarify the efficacy of various adjuvant drugs combined with phototherapy for neonatal hyperbilirubinemia. Fibrates and UDCA ranked higher in the analysis, while probiotics, zinc, and agar showed modest effects, and phenobarbital showed no clear benefit. Adjunctive pharmacological therapies may be associated with reductions in bilirubin levels when combined with phototherapy. However, given the limited long-term safety data, restricted geographical representation of included studies, and potential limitations in drug availability across different regions, these findings should be interpreted cautiously. Further high-quality randomized controlled trials are required before clinical application.

## Figures and Tables

**Table 1:** Characteristics of the studies.

ID	Study	Country	Sample	Sex (Female/Male)	Gestational Age (Week)	Initial Bilirubin (mg/dL)	Adjuvant Drugs	Group Sample	Outcome Reported
1	M. L. Tsai 2022 [9]	China	83	20/23	38.0 ± 0.20	16.78 ± 0.41	probiotic	43	bilirubin levels at 24 h; phototherapy duration
				25/15	37.88 ± 0.18	16.13 ± 0.41	phototherapy only	40	
2	P. Kumar 2017 [24]	India	90	21/24	38.7 ± 1.2	18 ± 2.1	clofibrate	45	bilirubin levels at 24, 48 h; phototherapy duration
				22/23	38.2 ± 1.2	18.6 ± 2.0	phototherapy only	45	
3	A. Shah Farhat 2024 [12]	Iran	80	NR	36.4 ± 2.39	17.52 ± 1.54	phenobarbital	40	bilirubin levels at 24 h; phototherapy duration; admission duration
				NR	36.9 ± 2.16	17.84 ± 1.41	phototherapy only	40	
4	E. Babaie 2023 [37]	Iran	120	20/20	NR	16.67 ± 2.08	ursodeoxycholic acid	40	bilirubin levels at 24 h
				27/13	NR	17.34 ± 2.15	probiotic	40	
				17/23	NR	17.28 ± 2.23	phototherapy only	40	
5	Y. Zahedpasha 2007 [8]	Iran	60	14/16	NR	18.21 ± 1.85	clofibrate	30	bilirubin levels at 24, 48 h
				14/16	NR	17.50 ± 2.34	phototherapy only	30	
6	T. H. Mandlecha 2023 [21]	India	106	26/27	NR	19.11 ± 2.16	zinc	53	bilirubin levels at 24, 48 h
				30/23	NR	18.57 ± 2.144	phototherapy only	53	
7	S. Ahmadipour 2019 [40]	Iran	83	NR	39.20 ± 3.90	NR	probiotic	40	admission duration
				NR	39.00 ± 4.10	NR	phototherapy only	43	
8	N. Honar 2016 [28]	Iran	80	21/19	NR	15.9 ± 1.7	ursodeoxycholic acid	40	bilirubin levels at 24, 48 h; phototherapy duration
				22/18	NR	16.3 ± 1.5	phototherapy only	40	
9	P. Khoshnevisasl 2020 [26]	Iran	112	27/29	38.8 ± 1.03	NR	zinc	56	bilirubin levels at 24, 48 h; admission duration
				31/25	38.8 ± 0.98	NR	phototherapy only	56	
10	H. Nassif 2024 [19]	Pakistan	72	20/16	33.5 ± 1.71	16.68 ± 1.69	probiotic	36	admission duration
				14/22	33.62 ± 1.95	16.92 ± 1.63	phototherapy only	36	
11	M.-L. Tsai 2025 [10]	China	277	46/43	37.93 ± 0.12	15.84 ± 0.25	Probiotic (AP-32)	89	admission duration
				41/55	37.99 ± 0.12	16.08 ± 0.23	Probiotic (CP-9)	96	
				51/41	38.08 ± 0.12	15.67 ± 0.23	phototherapy only	92	
12	O. Serce 2014 [14]	Turkey	119	26/12	37.7 ± 1.7	13.6 ± 2.9	probiotic	58	bilirubin levels at 24, 48 h; admission duration
				29/32	37.9 ± 2.02	13.6 ± 2.4	phototherapy only	61	
13	S. M. Abdel-Aziz Ali 2022 [41]	Egypt	60	13/17	38.10 ± 2.11	16.70 ± 1.58	agar	30	bilirubin levels at 24 h; phototherapy duration
				14/16	37.80 ± 1.63	17.20 ± 1.27	phototherapy only	30	
14	N. Rana 2011 [16]	India	286	NR	NR	NR	zinc	145	phototherapy duration
				NR	NR	NR	phototherapy only	141	
15	G. Faal 2020 [33]	Iran	60	NR	33.2 ± 1.27	12.2 ± 71.67	zinc	30	bilirubin levels at 24 h; phototherapy duration
				NR	32.1 ± 1.77	10.2 ± 35.24	phototherapy only	30	
16	M. M. Gharehbaghi 2020 [31]	Iran	120	NR	37.75 ± 4.93	19.88 ± 2.33	ursodeoxycholic acid (5 mg/kg/dose, q12 h)	40	bilirubin levels at 24, 48 h
				NR	38.1 ± 1.05	19.33 ± 2.51	ursodeoxycholic acid (7.5 mg/kg/dose, q12 h)	40	
				NR	38.55 ± 1.01	19.76 ± 2.64	phototherapy only	40	
17	A. Ghorui 2024 [30]	India	80	21/19	NR	15 ± 3.19	calcium phosphate	40	bilirubin levels at 24 h; admission duration
				16/24	NR	16.1 ± 2.64	phototherapy only	40	
18	M. Zarkesh 2023 [7]	Iran	92	NR	NR	17.03 ± 1.81	ursodeoxycholic acid	49	bilirubin levels at 24, 48 h; admission duration
				NR	NR	17.49 ± 1.92	phototherapy only	43	
19	M. H. Awad 2021 [38]	Egypt	120	28/32	NR	19.4 ± 3.8	Fenofibrate (10 mg/kg/day for 1 day)	60	bilirubin levels at 24, 48 h; phototherapy duration
				28/32	NR	19.4 ± 4.4	Fenofibrate (10 mg/kg/day for 2 days)	60	
				36/24	NR	18.8 ± 4.1	phototherapy only	60	
20	S. K. Shabo 2023 [13]	Iraq	100	22/28	38.58 ± 0.758	19.28 ± 0.427	fenofibrate	50	bilirubin levels at 24, 48 h; admission duration
				29/21	38.52 ± 0.995	19.21 ± 0.518	phototherapy only	50	
21	S. H. Saadat 2023 [15]	Iran	86	22/21	38.19 ± 0.98	17.31 ± 1.56	fenofibrate	43	bilirubin levels at 24, 48 h; phototherapy duration; admission duration
				16/27	38.21 ± 1.25	17.61 ± 1.67	phototherapy only	43	
22	M. A. Kaabneh 2015 [27]	Jordan	200	52/51	39 ± 1	14.7 ± 1.6	phenobarbital	103	bilirubin levels at 24, 48 h;
				50/47	39 ± 1	14.6 ± 1.5	phototherapy only	97	
23	R. Fallah 2012 [32]	Iran	60	NR	38.23 ± 0.971	19.54 ± 3.07	clofibrate	30	bilirubin levels at 24, 48 h; phototherapy duration; admission duration
				NR	37.87 ± 1.07	19.5 ± 2.21	phototherapy only	30	
24	F. Eghbalian 2023 [36]	Iran	100	NR	NR	19.49 ± 1.17	clofibrate	50	admission duration
				NR	NR	18.13 ± 2.5	phototherapy only	50	
25	R. Sharafi 2010 [11]	Iran	60	NR	NR	17.24 ± 1.48	clofibrate	30	bilirubin levels at 24, 48 h; phototherapy duration
				NR	NR	17.42 ± 1.44	phototherapy only	30	
26	M. Habibi 2012 [29]	Iran	52	11/15	NR	20.788 ± 2.3852	clofibrate	26	bilirubin levels at 24 h;
				11/15	NR	20.523 ± 2.4458	phototherapy only	26	
27	M. Nikouei 2024 [18]	Iran	290	81/79	38.25 ± 1.053	16.65 ± 2.90	ursodeoxycholic acid	160	bilirubin levels at 24, 48 h; admission duration
				64/66	38.27 ± 1.092	16.36 ± 2.96	phototherapy only	130	
28	F. Eghbalian 2024 [35]	Iran	150	43/32	37.8 ± 0.8	NR	probiotic	75	bilirubin levels at 24, 48 h; phototherapy duration; admission duration
				39/36	37.6 ± 0.7	NR	phototherapy only	75	
29	A. Mahyar 2019 [22]	Iran	40	9/11	38.5 ± 2	18 ± 2.4	clofibrate	20	bilirubin levels at 24, 48 h;
				12/8	17.9 ± 3.1	39 ± 1	phototherapy only	20	
30	R. Akefi 2022 [39]	Iran	220	48/62	NR	16.8 ± 2.4	ursodeoxycholic acid	110	bilirubin levels at 24 h; phototherapy duration
				58/52	NR	15.7 ± 2.5	phototherapy only	110	
31	W. Liu 2015 [23]	China	68	NR	NR	20.29 ± 3.04	phenobarbital and probiotic	34	bilirubin levels at 24 h; phototherapy duration
				NR	NR	20.53 ± 2.81	phenobarbital	34	
32	S. A. H. Nouri 2022 [17]	Iran	194	51/46	NR	16.93 ± 1.43	probiotic	97	bilirubin levels at 24, 48 h;
				57/40	NR	16.73 ± 1.53	phototherapy only	97	
33	A. Kumar 2014 [25]	India	80	28/12	37.6 ± 1.5	13.9 ± 2.5	zinc	40	bilirubin levels at 48 h; phototherapy duration
				25/15	37.7 ± 1.4	13.4 ± 1.9	phototherapy only	40	
34	M. S. Elfarargy 2021 [34]	Egypt	200	54/46	35.7 ± 0.6	17.7 ± 1.1	zinc	100	bilirubin levels at 48 h;
				58/42	35.8 ± 0.5	17.6 ± 1.2	phototherapy only	100	
35	M. Mosharref 2021 [20]	Bangladesh	60	10/20	38.1 ± 1.8	17.19 ± 1.98	fenofibrate	30	bilirubin levels at 24, 48 h; admission duration
				13/17	37.8 ± 1.1	17.02 ± 2.26	phototherapy only	30	

Note: NR means not reported.

**Table 2:** Ranking of adjuvant drugs.

Adjuvant Drugs	Bilirubin Levels at 24 h		Bilirubin Levels at 48 h		Phototherapy Duration		Admission Duration	
	SUCRA	PbBT	SUCRA	PbBT	SUCRA	PbBT	SUCRA	PbBT
phototherapy only	0.10	0.00%	0.09	0.00%	0.38	0.03%	0.28	0.00%
clofibrate	0.78	13.89%	0.91	60.88%	0.27	0.85%	0.84	40.83%
ursodeoxycholic acid	0.71	6.43%	0.56	3.77%	0.62	13.36%	0.82	32.72%
calcium phosphate	0.84	53.89%	NA	NA	NA	NA	0.36	2.61%
probiotics	0.32	0.06%	0.37	1.46%	0.53	8.9%	0.50	0.59%
fenofibrate	0.51	1.43%	0.56	3.83%	0.73	25.9%	0.79	20.8%
agar	0.58	12.86%	NA	NA	0.59	19.68%	NA	NA
phenobarbital	0.28	0.21%	0.71	29.59%	0.39	3.55%	0.07	0.23%
zinc sulfate	0.51	4.29%	0.31	0.47%	0.43	3.31%	0.33	2.23%
Phenobarbital& probiotics	0.38	6.94%	NA	NA	0.55	24.42%	NA	NA

Note: SUCRA means surface under the cumulative ranking curve. NA means not applicable. PbBT means probability of being the best treatment.

**Table 3:** Safety data of included studies.

ID	Study	Adjuvant Drugs	Group Sample	Adverse Events	Blood Transfusion	Mortality
1	M. L. Tsai 2022 [9]	probiotic	43	None	None	None
		phototherapy only	40	None	None	None
2	P. Kumar 2017 [24]	clofibrate	45	None	NA	None
		phototherapy only	45	None	4	None
3	A. Shah Farhat 2024 [12]	phenobarbital	40	NA	NA	NA
		phototherapy only	40	NA	NA	NA
4	E. Babaie 2023 [37]	ursodeoxycholic acid	40	None	NA	None
		probiotic	40	5 mild abdominal pain	NA	None
		phototherapy only	40	None	NA	None
5	Y. Zahedpasha 2007 [8]	clofibrate	30	None	None	None
		phototherapy only	30	None	None	None
6	T. H. Mandlecha 2023 [21]	zinc	53	None	NA	None
		phototherapy only	53	None	NA	None
7	S. Ahmadipour 2019 [40]	probiotic	40	None	NA	None
		phototherapy only	43	None	NA	None
8	N. Honar 2016 [28]	ursodeoxycholic acid	40	None	NA	None
		phototherapy only	40	None	NA	None
9	P. Khoshnevisasl 2020 [26]	zinc	56	None	NA	None
		phototherapy only	56	None	NA	None
10	H. Nassif 2024 [19]	probiotic	36	1 case of rash, 1 case of diarrhea, and 1 case of unstable body temperature	NA	None
		phototherapy only	36	3 case of rash, 1 case of diarrhea, and 3case of unstable body temperature	NA	None
11	M.-L. Tsai 2025 [10]	Probiotic (AP-32)	89	None	NA	None
		Probiotic (CP-9)	96	None	NA	None
		phototherapy only	92	None	NA	None
12	O. Serce 2014 [14]	probiotic	58	None	NA	None
		phototherapy only	61	None	NA	None
13	S. M. Abdel-Aziz Ali 2022 [41]	agar	30	None	NA	None
		phototherapy only	30	None	NA	None
14	N. Rana 2011 [16]	zinc	145	3 cases of diarrhea, 4 cases of vomiting, and 1 case of rash	NA	None
		phototherapy only	141	1 case of diarrhea, 6 cases of vomiting	NA	None
15	G. Faal 2020 [33]	zinc	30	None	NA	None
		phototherapy only	30	None	NA	None
16	M. M. Gharehbaghi 2020 [31]	ursodeoxycholic acid (5 mg/kg/dose, q12h)	40	None	NA	None
		ursodeoxycholic acid (7.5 mg/kg/dose, q12 h)	40	None	NA	None
		phototherapy only	40	None	NA	None
17	A. Ghorui 2024 [30]	calcium phosphate	40	None	NA	None
		phototherapy only	40	None	NA	None
18	M. Zarkesh 2023 [7]	ursodeoxycholic acid	49	None	NA	None
		phototherapy only	43	None	NA	None
19	M. H. Awad 2021 [38]	Fenofibrate (10 mg/kg/day for 1 day)	60	1 case of anemia and 1 case of leukopenia; 8 cases of abdominal distension and diarrhea; 4 cases of elevated liver enzymes	1	None
		Fenofibrate (10 mg/kg/day for 2 days)	60	1 case of anemia, 6 cases of abdominal distension and diarrhea, and 3 cases of elevated liver enzymes	1	None
		phototherapy only	60	3 cases of anemia; 1 case of leukopenia; 8 cases of abdominal distension and diarrhea; 4 cases of elevated liver enzymes	3	None
20	S. K. Shabo 2023 [13]	fenofibrate	50	None	NA	None
		phototherapy only	50	None	NA	None
21	S. H. Saadat 2023 [15]	fenofibrate	43	None	NA	None
		phototherapy only	43	None	NA	None
22	M. A. Kaabneh 2015 [27]	phenobarbital	103	NA	7	NA
		phototherapy only	97	NA	15	NA
23	R. Fallah 2012 [32]	clofibrate	30	1 case of gastrointestinal reaction	NA	None
		phototherapy only	30	None	NA	None
24	F. Eghbalian 2023 [36]	clofibrate	50	None	NA	None
		phototherapy only	50	None	NA	None
25	R. Sharafi 2010 [11]	clofibrate	30	None	NA	None
		phototherapy only	30	None	NA	None
26	M. Habibi 2012 [29]	clofibrate	26	None	NA	None
		phototherapy only	26	None	NA	None
27	M. Nikouei 2024 [18]	ursodeoxycholic acid	160	None	NA	None
		phototherapy only	130	None	NA	None
28	F. Eghbalian 2024 [35]	probiotic	75	None	NA	None
		phototherapy only	75	None	NA	None
29	A. Mahyar 2019 [22]	clofibrate	20	None	NA	None
		phototherapy only	20	1 case of rash	NA	None
30	R. Akefi 2022 [39]	ursodeoxycholic acid	110	None	NA	None
		phototherapy only	110	None	NA	None
31	W. Liu 2015 [23]	phenobarbital and probiotic	34	None	NA	None
		phenobarbital	34	None	NA	None
32	S. A. H. Nouri 2022 [17]	probiotic	97	None	NA	None
		phototherapy only	97	None	NA	None
33	A. Kumar 2014 [25]	zinc	40	3 cases of vomiting; 3 cases of rash; 4 cases of diarrhea	None	None
		phototherapy only	40	2 cases of vomiting; 3 cases of rash; 3 cases of diarrhea	None	None
34	M. S. Elfarargy 2021 [34]	zinc	100	None	NA	None
		phototherapy only	100	None	NA	None
35	M. Mosharref 2021 [20]	fenofibrate	30	None	NA	None
	M. L. Tsai 2022 [9]	phototherapy only	30	None	NA	None

NA means not applicable.

## Data Availability

All data necessary for interpreting the meta-analysis are available in the main text or the Supplementary Materials.

## Supplementary Materials

The following supporting information can be downloaded at: https://www.mdpi.com/article/10.3390/children13040573/s1.

## Abbreviations

The following abbreviations are used in this manuscript:

UDCA	ursodeoxycholic acid
RCTs	randomized controlled trials
MD	mean difference
SD	standard deviation
SUCRA	surface under the cumulative ranking curve
PbBT	probability of being the best treatment
